# The effects of waxy barley on defecation, sleep, mental health, and quality of life: a randomized double-blind parallel-group comparison study

**DOI:** 10.1186/s40101-025-00393-8

**Published:** 2025-05-07

**Authors:** Mari Honda, Satomi Minato-Inokawa, Kimie Matsuura, Ayaka Ito, Yuko Nitta, Daisuke Kimura, Yutaka Yoshikawa

**Affiliations:** 1 Department of Health, Sports, and Nutrition, Faculty of Health and Welfare, Kobe Women’s University, 4 - 7- 2 Minatojima-Nakamachi, Chuo-Ku, Kobe City, Hyogo, 650 - 0046 Japan; 2 Laboratory of Community Health and Nutrition, Department of Bioscience, Graduate School of Agriculture, Ehime University, 10 - 1 Dogohimata, Matsuyama City, Ehime, 790 - 0825 Japan

**Keywords:** Waxy barley, Dietary fiber, β-glucan, Defecation, Constipation, Sleep, PSQI, Mental health, Health-related quality of life (HRQoL)

## Abstract

**Background:**

Dietary fiber (DF) is beneficial for preventing constipation, and the metabolites produced by gut microbiota fermentation are suggested to positively influence on depression and sleep. Additionally, constipation has been reported to affect mental health and health-related quality of life (HRQoL). This study aimed to increase DF intake and examined its effects on daily DF and β-G consumption using two types of waxy barley (WB), rich in DF with varying β-glucan (β-G) content. Additionally, this study examined the effects of WB consumption on defecation, sleep, mental health, and HRQoL.

**Methods:**

A randomized double-blind parallel-group comparison study was conducted on 68 young Japanese women, using Kirarimochi (Group K) as common WB cultivar and Fukumi Fiber (Group F) as high-β-G WB cultivar. Participants consumed WB rice for 4 weeks, targeting 3 g/day of β-G (48 g/day of WB). We estimated the intake of WB and DF including β-G from the daily records. Defecation was evaluated through daily records and Rome IV criteria-based surveys. Sleep, mental health, and HRQoL were evaluated using PSQI-J, J-PHQ-9, and SF-36, respectively.

**Results:**

Both groups consumed about 40 g/day of WB. DF and β-G intakes from barley were 6.3 g/2.5 g in Group K and 10.7 g/4.3 g in Group F. Regarding defecation, both groups showed increases defecation days, defecation frequency and stool amount, with no differences between groups. Regarding sleep, Group F showed a decrease (improvement) in the PSQI global score, with improvements noted between groups. Regarding mental health, both groups showed decreases (improvements) in the PHQ-9 score, with Group F showing improvement between groups. Regarding HRQoL, summary scores showed improvements: physical health in Group K and mental health in Group F.

**Conclusion:**

To increase β-G intake, high-β-G WB cultivars are effective; however, WB cultivars overall can potentially serve as excellent sources of DF. Effects on defecation may be expected not only from high-β-G WB but also from common WB with β-G intake below the target of 3 g/day. Although high β-G WB may have more beneficial for sleep and mental health, additional studies are required.

## Background

The intake of dietary fiber (DF) is known to have positive effects on bowel movements, weight management, carbohydrate and lipid metabolism, and the gut microbiota. It is also beneficial for preventing cardiovascular diseases, type 2 diabetes, and colorectal cancer [1, 2]. Furthermore, insufficient DF intake has been suggested to also be associated with chronic inflammation and depression [1]. However, according to the Global Burden of Disease Study [3], which surveyed 195 countries, DF intake in 2017 was below 20 g/day in most regions, with especially low levels observed in Asia. Japanese adults’ DF intake averaged over 20 g/day in the 1950 s, but during the high economic growth period of the 1960 s, it significantly decreased [4, 5]. According to 2019 statistics [6], DF intake of men and women in their 20 s to 50 s was below the dietary reference intakes (20–21 g/day for adult men and 17–18 g/day for adult women, Ministry of Health, Labour and Welfare, 2020 edition) [7], especially, with women in their 20 s having the lowest intake among adults, averaging 14.6 g [6].

On the other hand, regarding constipation closely associated with insufficient DF, the Global Study [8], which investigated the prevalence of functional gastrointestinal disorders (FGID) across 33 countries on six continents, reported that the prevalence of functional constipation (FC) without organic disease among Japanese individuals was 16.6%, a comparatively high rate compared to other countries. Additionally, globally, functional constipation (FC) is more common in women than in men [8]. In Japan, the prevalence of constipation (per 1000 population) also sharply increases among women, with rates of 16.9 compared to 4.1 for men aged 15–19 years [9]. It is known that Japanese women maintain high levels of constipation prevalence even after their 20 s, indicating a more severe condition. Reviews on constipation have reported that it affects mental and physical aspects of quality of life (QoL), with an impact comparable to other common chronic diseases [10]. Even among Japanese individuals, reduced health-related quality of life (HRQoL) scores and impacts on work productivity and activity have been reported [11]. Furthermore, the health burden is reported to be greater than that of conditions like type 2 diabetes mellitus (T2DM) and irritable bowel syndrome (IBS) [12]. Considering constipation and its associated impacts, it is important to examine dietary approaches that contribute to its prevention and improvement.

In this study, we focused on waxy barley as a means to increase DF intake to aid in constipation management. In recent years in Japan, waxy barley has gained attention for its good texture and nutritional value, especially its high DF content, and waxy barley rice, made by cooking whole grains mixed with rice, has become increasingly popular [13]. Barley is one of the most important grains cultivated worldwide, rich in DF alongside oats, and contains a higher proportion of β-glucan (β-G), a type of soluble DF, compared to other major cereals [14, 15]. Additionally, waxy barley refers to a type of barley with very low or no amylose content in its starch and contains higher levels of β-G compared to non-waxy barley [13, 16]. Therefore, for Japanese people whose staple food is rice, consuming waxy barley rice could serve as an excellent source of DF by providing an affordable and consistent intake of waxy barley in their daily meals, potentially contributing to constipation management.

Barley and barley-derived β-G are known for various physiological functionalities, and both the European Food Safety Authority (EFSA) and Health Canada have approved health claims regarding their cholesterol-lowering effects [17–19]. Additionally, the benefits of barley and barley-derived β-G on lipid and carbohydrate metabolism, including the suppression of postprandial blood glucose levels [20, 21], have been widely reported. Furthermore, studies have been advancing on their effects on immunity and inflammation, as well as the composition and metabolic products of the gut microbiota [22]. However, although the impact of DF on constipation is perhaps its best-known benefit [1], the effect of barley consumption on constipation has been examined in very few studies. Neither past nor recent meta-analyses on the effects of DF on constipation have included studies using barley or β-glucan [23, 24].

Additionally, DF is known to influence the microbiota-gut-brain axis [25], a bidirectional system linking the gut, its microbiota, and the brain through various pathways [26–28]. It is known that one of these interactions involves short-chain fatty acids (SCFAs) like acetate, propionate, and butyrate, which are produced by the fermentation of DF by gut microbiota, playing a role in regulating various bodily functions including neurotransmitter synthesis and immune-inflammatory responses [29]. Furthermore, several human studies have reported that SCFAs are associated with anxiety and depressive symptoms [30, 31]. The first systematic review investigating the association between SCFA concentrations and mood disorders [32] reported that although the mechanisms and direction of effects remain unclear, SCFAs might be involved in depressive symptoms. Additionally, regarding sleep, it has also been suggested that SCFAs may positively influence sleep by acting on serotonin and GABA production [33]. There have already been numerous positive reports from animal studies on the effects of barley administration on SCFAs, and some human studies have also reported increases in fecal SCFA concentrations with the intake of foods containing barley or barley-derived β-G [34–36]. Therefore, the intake of barley has the potential to positively impact not only bowel movements but also sleep and mental health, including depressive symptoms. However, there are very few reports examining the relationship between barley consumption and sleep or mental health in humans.

Therefore, this study conducted an intervention trial targeting young women, who face significant DF insufficiency and a rapid increase in constipation, using two types of waxy barley with different β-G contents. In Japan, several domestic varieties of waxy barley have been developed. One of these includes a cultivar of waxy barley, developed to have about 1.5 times higher β-G content compared to conventional non-waxy barley, which is now extensively grown throughout Japan [37]. Subsequently, a waxy barley cultivar with more than twice the β-G content of conventional waxy barley was developed [38]. In this study, participants cooked one of the two types of waxy barley at home and consumed it as waxy barley rice aiming for a daily intake of 3 g of β-G. This study aimed to examine, within an intervention trial conducted as part of regular meals, how differences in waxy barley cultivars with varying β-G content affect the intake of β-G and DF as well as their impacts on defecation, sleep, mental health, and HRQOL. These results may offer insights into effective methods for increasing DF intake, which is often insufficient, the effects of barley consumption on defecation, and the potential health benefits of barley other than the known lipid and carbohydrate metabolism.

## Methods

### Study design and participants

**Fig. 1:**
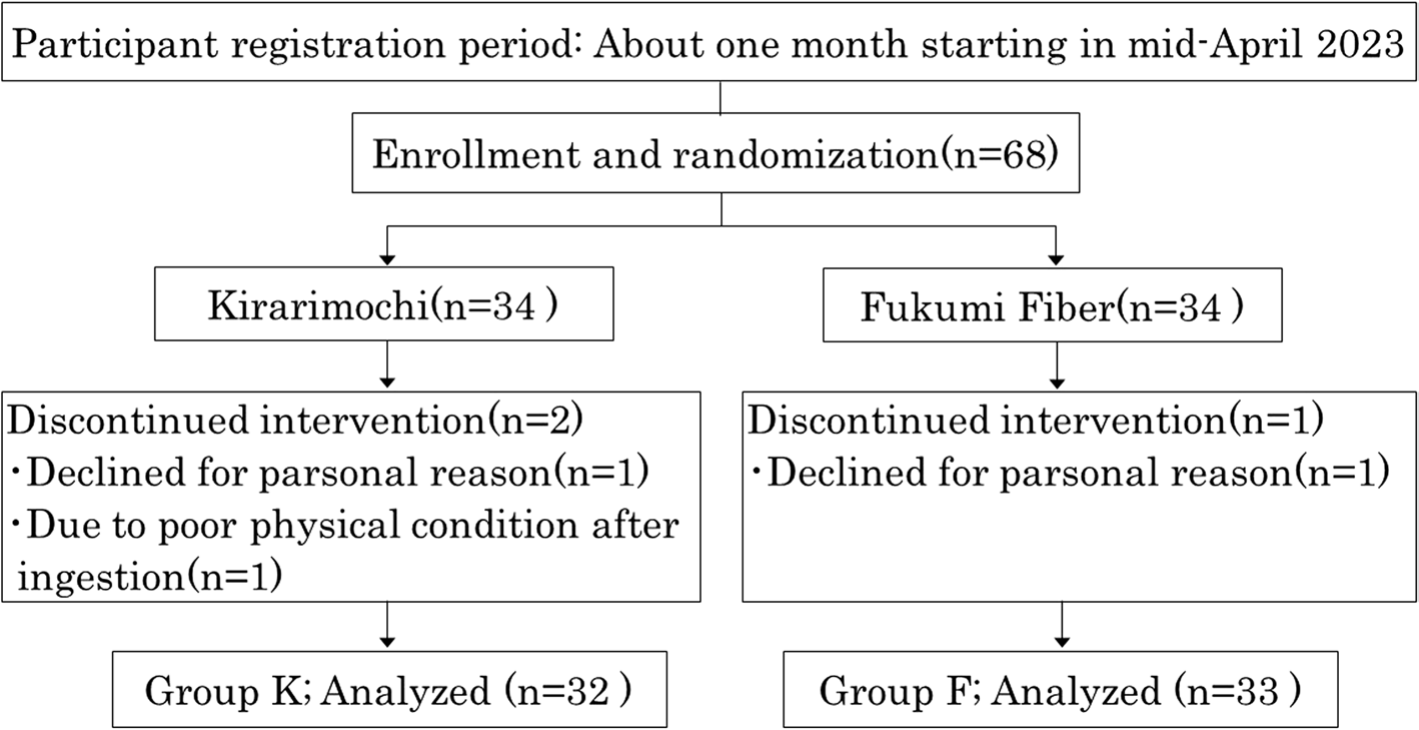
Selection of participants

We conducted a randomized double-blind parallel-group comparison study on healthy young Japanese female college students. In this study, two waxy barleys were used as the test food: “Kirarimochi,” a common cultivar of waxy barley currently distributed in Japan, and “Fukumi Fiber,” a waxy barley cultivar extremely rich in β-G and DF. The intake period was four weeks beginning June 3, 2023, with a pre-intervention baseline study period of two weeks before intake. We enrolled 68 healthy female participants aged 18–22 and, using a computer-generated permuted block method, divided them into two groups: Group K (34 participants consuming the Kirarimochi cultivar of waxy barley) and Group F (34 participants consuming the Fukumi Fiber cultivar of waxy barley) (Fig. 1). Exclusion criteria for participants were (1) those with barley allergy; (2) those with wheat allergy, considering cross-reactivity; and (3) those who regularly used medications or supplements for bowel movements, sleep, or mental health.

The protocol for this study was approved by the Research Ethics Committee for Human Participants of Kobe Women’s University and Kobe Women's Junior College (Approval No. 2022–41 - 1). Participants were informed in advance of the purpose, methods, risks, and handling of personal information of the study, and their written consent was obtained.

### Test foods and intake methods

The test foods used were “Kirarimochi” (from Okayama Prefecture, Japan), a naked barley cultivar of two-row barley, used as a common waxy barley cultivar, and “Fukumi Fiber” (from Okayama Prefecture, Japan), a naked barley cultivar of six-row barley, used as a high-β-G waxy barley cultivar. Both are waxy barley varieties that contain higher amounts of β-G than non-waxy barley [13, 16]. Additionally, although typical barley cultivars have caryopsis with adhering hulls at maturity, known as covered (hulled) barley, the cultivars employed in this research are both a variant called naked (hulless) barley. Naked barley does not require extensive polishing to remove the hull, allowing it to retain more nutrients, including starch, protein, and β-G, in the barley grains [39, 40]. Kirarimochi was registered as a cultivar by the Ministry of Agriculture, Forestry and Fisheries (MAFF) in 2009. It is a domestic barley cultivar with a higher β-G content compared to existing naked barley cultivars, and it has been confirmed to have a high preference among Japanese people [13, 17]. It is now widely cultivated across various regions of Japan. Fukumi Fiber, registered as a cultivar in 2019, is a domestic barley cultivar with extremely high β-G content and excellent grain quality [38]. Presently, it is grown in western Japan, with a focus on Hyogo and Okayama Prefectures.

In this study, we set a target intake of 3 g/day, the amount of β-G that has been reported to have multiple clinical benefits and is recognized by EFSA and FDA as necessary to achieve specific health effects [41, 42]. Additionally, the ratio of waxy barley mixed with rice was determined to be 30% considering the participants’ acceptance, and the daily intake was designed to be 48 g of dried waxy barley (112 g of polished rice). When a certain amount (48 g/day) of each type of waxy barley was consumed, the amount of β-G from barley was 2.93 g/day for Kirarimochi and 5.14 g/day for Fukumi Fiber, and the total DF amount was 7.39 g/day and 12.67 g/day, respectively (Table 1). The content of Insoluble DF, high molecular weight soluble DF, low molecular weight soluble DF, and β-G in each waxy barley was quantified using the AOAC 2011. 25 method at the Japan Food Research Laboratories.

**Table 1:**
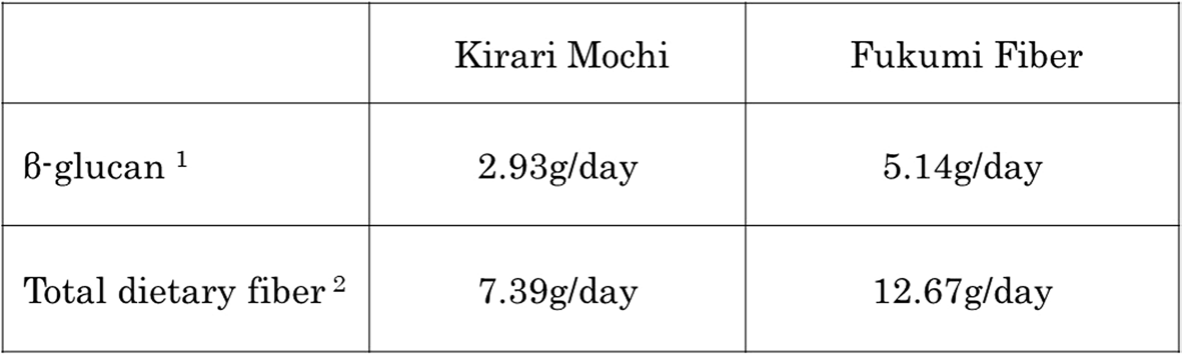
Amount of β-glucan and dietary fiber contained when consuming a fixed amount (48 g/day) of each type of waxy barley

^1^Analyzed by Japan Food Research Laboratories using (1 3), (1 4) β-glucan measurement kit (made by Megazyme)

^2^Analyzed by Japan Food Research Laboratories, using AOAC 2011.25 method

We packaged the amount of waxy barley per cup of rice so that it could be cooked at home with a 30% waxy barley mixture and distributed dried waxy barley that could not be identified by appearance. The packaged waxy barley was marked with an alphabet that did not identify the cultivar it was, and its identity was also blinded to the research staff who were dealing with the participants. The participants mixed the distributed waxy barley with rice in a fixed ratio and cooked it, consuming the prepared waxy barley rice for 4 weeks. The participants needed to consume approximately 370 g/day of waxy barley rice to consume a certain amount of waxy barley (48 g/day). Therefore, we explained that participants should replace their habitual staple food with waxy barley rice and consume approximately 185 g of waxy barley rice at least twice per day. In addition, we provided participants with a special bowl to guide the amount of waxy barley rice per meal, ensuring consistent intake. Participants were instructed to maintain their usual dietary habits without making any changes, including altering their eating habits or starting new supplements, during the 4-week period of consuming the test food.

### Survey schedule and content

**Fig. 2:**
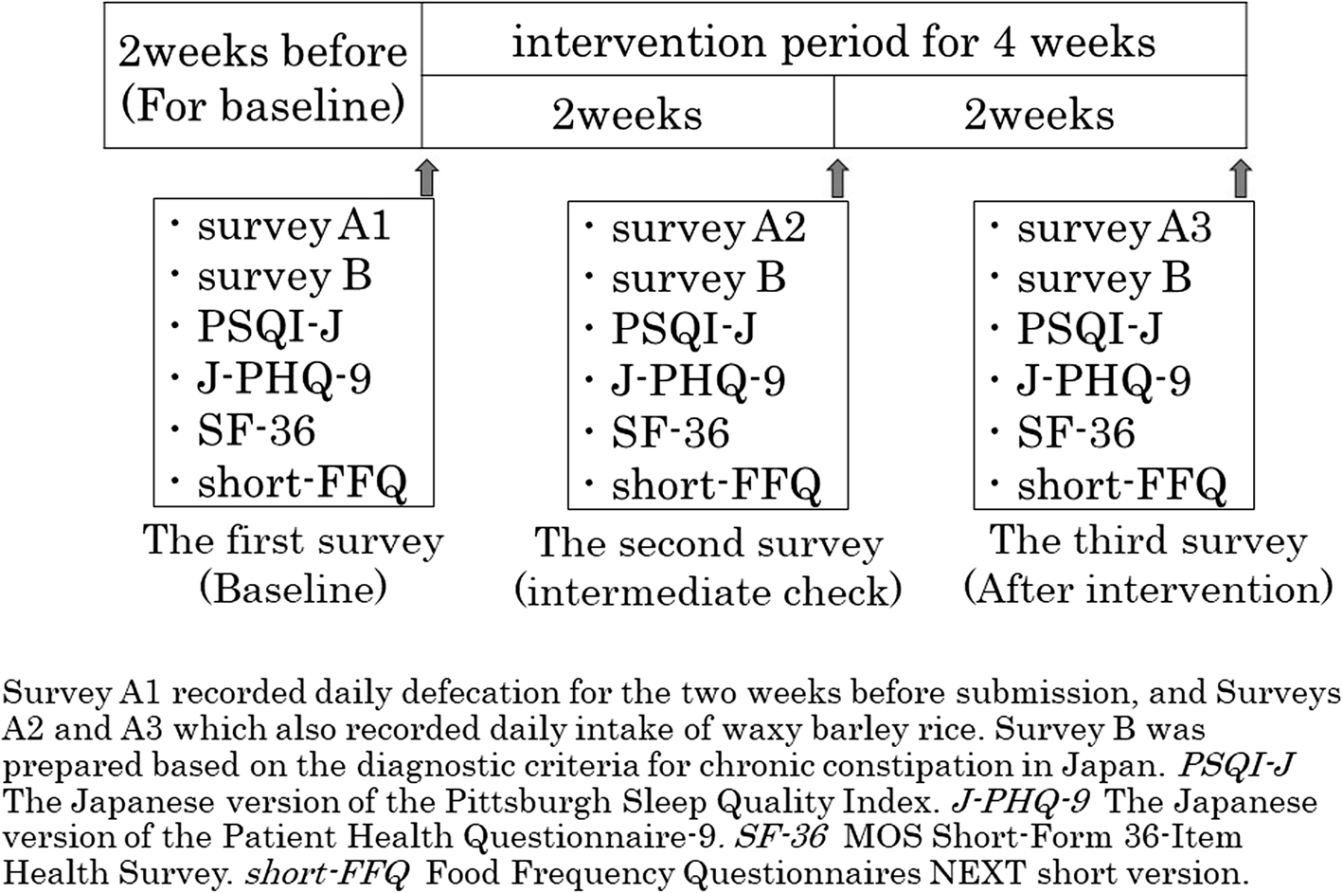
Study schedule and contents

The study schedule consisted of the first survey, the second survey, and the third survey (Fig. 2). The first survey was conducted during the two weeks before the intervention and served as the baseline data for the participants. Next, during the 4-week period of consuming waxy barley rice, the second survey and the third survey were conducted. The second survey covered the first two weeks of the four-week intervention period and included checking the recording status of the survey during the intervention. The third survey covered the latter 2 weeks of the intervention period. The survey content included the following six surveys: (1) Survey A (A1, A2, A3), (2) Survey B, (3) the Japanese version of the Pittsburgh Sleep Quality Index (PSQI-J), (4) the Japanese version of the Patient Health Questionnaire- 9 (J-PHQ- 9), (5) the Medical Outcomes Study 36-Item Short-Form Health Survey version 2 (SF- 36), and (6) the Food Frequency Questionnaires NEXT short version (short-FFQ). Survey A was a daily recording survey conducted during the 2 weeks prior to each submission, while the other five surveys were conducted three times during the first survey, the second survey, and the third survey.

Survey A1, referencing existing studies [43, 44], was created to record daily defecation status over the two weeks before submission. Surveys A2 and A3 were created to record daily intake of waxy barley rice in addition to defecation status. Based on the defecation records, the average number of defecation days (defecation days/survey days), defecation frequency (defecation frequency/defecation days), and stool amount (egg equivalents/defecation days) were calculated. In addition, based on the seven stool types recorded using the Bristol Stool Form Scale (BS) [45, 46], we categorized BS types 1 and 2 as clearly hard stools; BS types 3, 4, and 5 as typical stools; and BS types 6 and 7 as clearly loose stools [44], and calculated their respective percentages. For difficulty in defecation, the percentages were calculated for three patterns of subjective scales: no, slightly, and yes. In surveys A2 and A3 on waxy barley rice consumption, participants were asked to record the number of bowls of waxy barley rice consumed at each meal, and the amounts of total DF, insoluble DF, high molecular weight soluble DF, low molecular weight soluble DF, and β-G derived from waxy barley were calculated.

Survey B was prepared based on the diagnostic criteria for chronic constipation in Japan [47], which adopted the international criteria Rome IV [46], and if two or more of the six items (a–f) were checked, the participant was diagnosed with constipation (Table 2). In addition, if there was at least one symptom, it was considered as presence of constipation symptoms. In this study, for comparison within the group, the percentages of constipation, constipation symptoms, and each symptom (a–f), were analyzed before and after the intervention. For comparison between groups, changes in constipation, constipation symptoms, and each symptom (a–f) were classified into three levels: improvement, unchanged, and worsening. The quantified scores of these changes were then utilized for analysis.

**Table 2:**
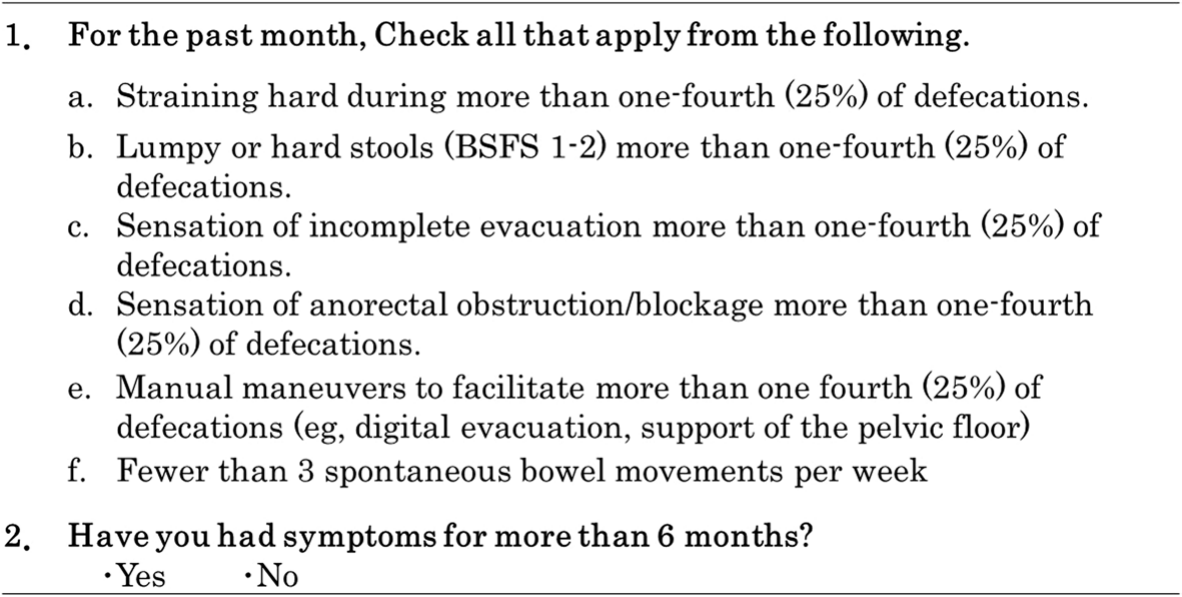
Survey on constipation symptoms (Survey B)

Based on diagnostic criteria for chronic constipation in Japan made according to Roma IV

The PSQI-J is used to evaluate subjective sleep quality over a one-month period and consists of seven component scores (sleep quality, sleep onset latency, sleep duration, sleep efficiency, sleep disturbances, use of sleep medication, and daytime dysfunction) [48, 49]. In this study, the seven component scores (range, 0–3) and the PSQI global score (range, 0–21) which is the sum of these scores, were calculated and used in the analysis to evaluate sleep status. Higher overall scores indicate lower subjective sleep quality, and the optimal PSQI-J cut-off value for sensitivity and specificity in Japanese is reported to be 5.5 [49].

The J-PHQ- 9 is a questionnaire consisting of nine items extracted from the Patient Health Questionnaire (PHQ), a self-administered version of PRIME-MD, which was developed for evaluating mental disorders in primary care [50, 51]. It assesses mental and physical conditions over a 2-week period, and its Japanese version has been proven effective for evaluating the severity of depression [50]. In this study, the scores for nine items (range, 0–3) and the PHQ- 9 score (range, 0–27) were calculated and used in the analysis to evaluate mental health. Higher scores indicate more severe symptoms of depression [51].

The SF- 36 is a 36-item self-administered questionnaire designed to measure comprehensive health concepts by eight areas as a survey of HRQoL, and has been validated validly in the Japanese version [52–54]. The eight areas are (1) physical functioning, (2) role physical, (3) bodily pain, (4) general health, (5) vitality, (6) social functioning, (7) role emotional, and (8) mental health. In this study, a questionnaire was used that assessed the condition of the patients over a one-month period. For the analysis, subscale scores were used, with scores for the eight domains (range 0–100) recalculated based on the Japanese national norms. In addition, three summary scores obtained from the Japanese version: physical component summary, mental component summary, and role/social component summary [54] were also used in the analysis.

The short-FFQ (Kenpakusha, Tokyo, Japan) used in the dietary intake frequency survey is an FFQ that has also been verified for accuracy against the long version of the FFQ used in the Next Generation Multi-purpose Cohort Study in Japan [55, 56]. The analysis utilized the intake of energy and nutrients, along with the intake of food groups, calculated based on the Standard Tables of Food Composition in Japan (2020 Edition) from the one-month dietary intake status obtained through the short-FFQ.

For responses to the PSQI-J, SF- 36, and short-FFQ, although the second and third surveys were conducted every 2 weeks, participants were asked to respond to the PSQI-J, SF- 36, and short-FFQ according to their conditions over the past month, as per the survey periods of each questionnaire.

### Side effects and discontinuation of the study

If gastrointestinal symptoms including diarrhea or other discomforts occur due to the consumption of the test food, and continued intake becomes difficult, the trial will be discontinued. In addition, if food allergy-like symptoms, including skin, mucous membrane, or gastrointestinal issues, occur, it is advised to stop intake immediately and recommend consultation with a medical institution.

### Statistical analyses

For the evaluation of waxy barley rice intake, data from Surveys A2 and A3, which recorded the number of cups of waxy barley rice consumed at each meal over a two-week period, were aggregated and used as one month’s consumption data for analysis. For the evaluation of defecation status, baseline data obtained from Survey A1, which was conducted 2 weeks before the intervention, was used, and one month’s defecation data aggregated from Surveys A2 and A3 served as the post-intervention data for analysis. For the evaluation of the frequency of constipation symptoms based on the Rome IV criteria, sleep status, mental health, HRQoL, and dietary intake, data from five surveys conducted during the first and third surveys (Survey B, PSQI-J, J-PHQ- 9, SF- 36, short-FFQ) were used. Based on these data, a baseline comparison between the two groups, a comparison of changes between the two groups before and after the intervention, and a within-group comparison before and after the intervention were conducted.

Statistical analyses were performed using SPSS version 27.0 (IBM SPSS Statistics, Japan). Since many of the data did not follow a normal distribution, variables other than the status of test food and dietary fiber intake were presented as the median and range (25 th and 75 th percentiles). For test food and dietary fiber intake data that followed a normal distribution, the mean and standard deviation were used for representation. The Unpaired *t* test or the Mann–Whitney *U* test was used to compare continuous data between the two groups. The Wilcoxon signed-rank sum test was used to compare within groups before and after the intervention. The chi-square test or Fisher’s exact test was used to analyze the rate of constipation symptoms and others. Additionally, using changes in constipation symptoms before and after the intervention based on survey B, a multivariable linear regression analysis was conducted. Changes in constipation, constipation symptoms and each symptom (a–f) were used as dependent variables, and four variables were used as independent variables: type of barley, frequency of waxy barley rice intake (%), days with waxy barley rice intake more than twice a day (%), and DF intake (either total DF, β-G, or insoluble DF). *P* values < 0.05 were considered significant.

## Results

Of the 68 participants in the study, two who did not submit surveys during the trial period and one who was suspected of experiencing health issues due to consumption of the test food withdrew from the study. Finally, 65 participants were included in the analysis, 32 in Group K and 33 in Group F (Fig. 1). One participant who experienced health issues reported itching during barley intake. The symptoms resolved after discontinuing consumption, but medical consultation was recommended.

### Comparison of baseline between groups before the intervention

The median age of Group K and Group F was both 20.0 years. The median Body Mass Index (BMI) was 20.4 in Group K and 21.6 in Group F. No differences in age or physical constitution were observed between the groups (Table 3). There were no differences between the two groups in defecation status during the 2 weeks before the intervention. In terms of mental health, only low self-esteem, which is a component of the J-PHQ- 9, showed a significant difference. However, there were no significant differences in all other sleep statuses, mental health, HRQoL, and dietary intake.

**Table 3:**
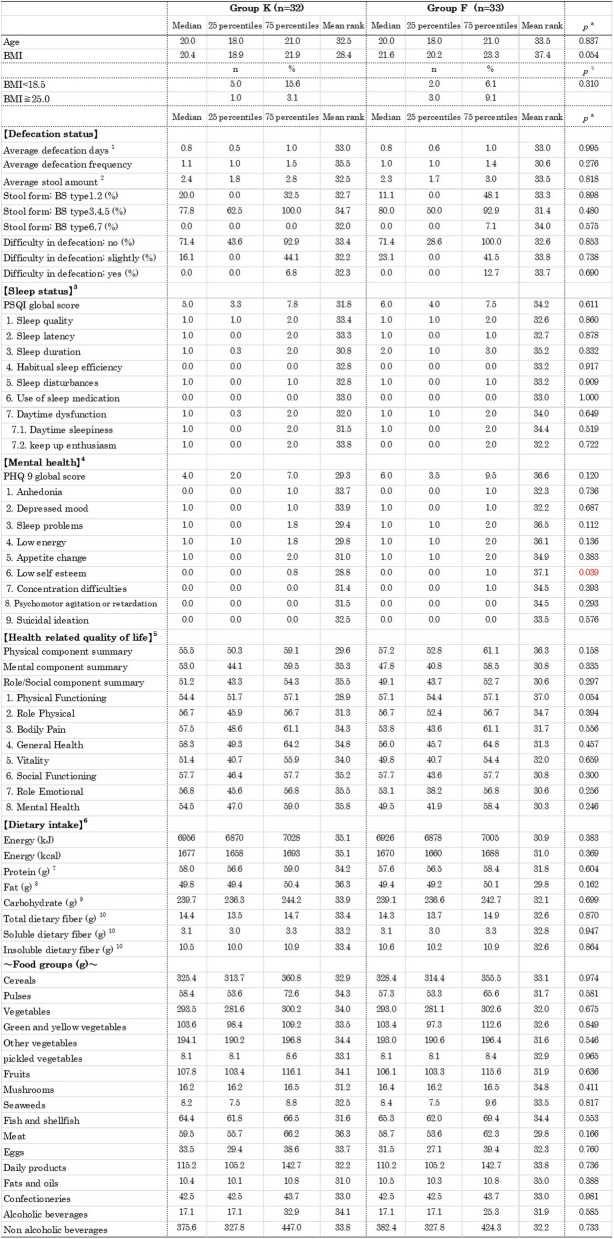
Comparison of baseline data between groups

^a^Mann Whitney U-test

^b^Chi-square test. Data were obtained from each survey (excluding Survey B) conducted as part of the first survey

^1^The data are averages per survey day. All other data for defecation status are averages per defecation day

^2^Number of egg conversion

^3^According to the Pittsburgh Sleep Quality Index (PSQI-J)

^4^According to the Patient Health Questionnaire- 9 (J-PHQ- 9)

^5^According to the Medical Outcomes Study 36-Item Short-Form Health Survey version 2 (SF- 36)

^6^According to the Food Frequency Questionnaires NEXT short version (short-FFQ)

^7^Protiens calculated based on the Japanese amino acid composition table

^8^Fatty acids expressed in triacylglycerol equivalents

^9^The amount of carbohydrates used for energy calculation based on the Standards Tables of Food Composition in Japan (2020 Edition)

^10^The dietary fiber intake was calculated using the modified Prosky method (enzymatic-gravimetric method) based on the Standard Tables of Food Composition in Japan (2020 Edition)

*BMI *Body Mass Index, *BS *Bristol stool form scale

### Status of test food and DF intake over 4 weeks

During the 4-week survey period, there were no significant differences in the days with waxy barley rice intake (%) and the frequency of waxy barley rice intake (%) (with a maximum of three servings per day) (Table 4). The median percentage of days with waxy barley rice intake more than twice a day (%) (with a barley weight of 48 g/day or more) was also 73.2% in Group K and 75.0% in Group F, with no significant difference between the two groups. Furthermore, the median daily barley intake was 40.7 g in Group K and 42.0 g in Group F, with no significant difference observed. Total DF intake (g) was significantly higher in Group F (10.7 ± 2.4 g) compared to Group K (6.3 ± 1.6 g) (*p* < 0.001). The intakes of insoluble DF, high molecular weight soluble DF, and low molecular weight soluble DF were also significantly higher in Group F, and the intake of β-G was also significantly higher in Group F (4.34 ± 0.97 g) than in Group K (2.48 ± 0.63 g) (*p* < 0.001). In addition, the percentage of individuals with an average β-G intake of 2.93 g or more was significantly higher in Group F (93.9%) than in Group K (21.9%) (*p* < 0.001).

**Table 4:**
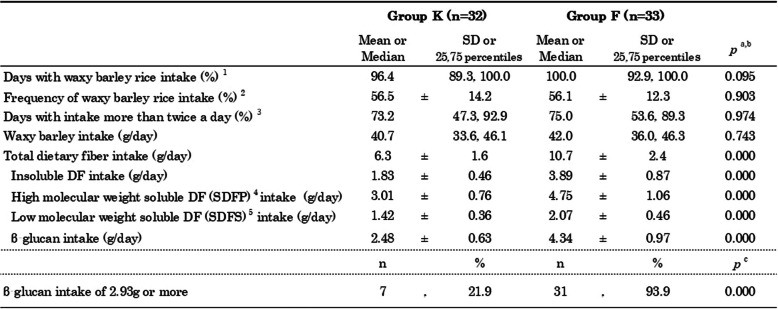
Status of test food and dietary fiber intake over 4 weeks

^a^Data expressed as means ± standard deviations (SD) were analyzed using Unpaired t-tests

^b^Data expressed as medians and ranges were analyzed using the Mann Whitney U test

^c^Data expressed as frequencies (n) and percentages (%) were analyzed using Fisher’s exact test

^1^The percentage represents the days with waxy barley rice intake, divided by the total number of survey days

^2^The percentage represents the frequency of waxy barley rice intake, divided by the number of survey days and three meals per day

^3^The percentage represents the days with intake more than twice a day, divided by the total number of survey days

^4^Soluble dietary fiber precipitated from 78% aqueous ethanol (using the AOAC 2011.25 method)

^5^Soluble dietary fiber that remains soluble in 78% aqueous ethanol (using the AOAC 2011.25 method)

*DF* Dietary fiber

### Comparison of changes between groups before and after the intervention

The comparison of changes between groups before and after the intervention is shown in Table 5.

**Table 5:**
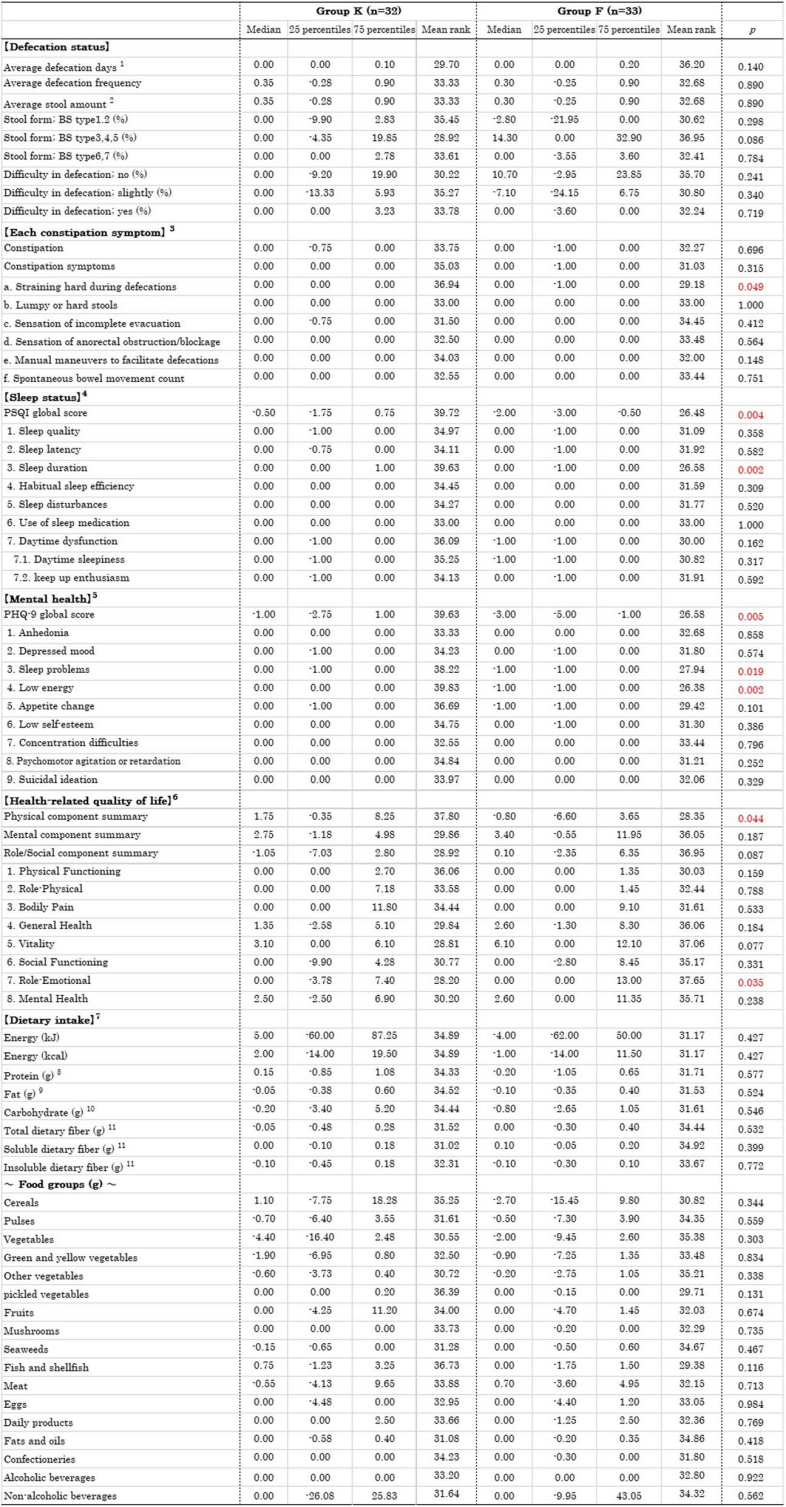
Comparison of changes between groups before and after the intervention

Using the Mann Whitney U-test. Changes in defecation status were analyzed using baseline data obtained from survey A1 and one month’s defecation data aggregated from Survey A2 and A3 (one-month data minus A1 data). For other comparisons, changes were analyzed using data from the first and third surveys (third survey data minus first survey data)

^1^The data are averages per survey day. All other data for defecation status are averages per defecation day

^2^Number of egg conversion

^3^The names of items a to f under each constipation symptom, based on the ROME IV criteria, are referenced in Survey B (Table 2)

^4^According to the Pittsburgh Sleep Quality Index (PSQI-J)

^5^According to the Patient Health Questionnaire- 9 (J-PHQ- 9)

^6^According to the Medical Outcomes Study 36-Item Short-Form Health Survey version 2 (SF- 36)

^7^According to the Food Frequency Questionnaires NEXT short version (short-FFQ)

^8^Protiens calculated based on the Japanese amino acid composition table

^9^Fatty acids expressed in triacylglycerol equivalents

^10^The amount of carbohydrates used for energy calculation based on the Standard Tables of Food Composition in Japan (2020 Edition)

^11^The dietary fiber intake was calculated using the modified Prosky method (enzymatic-gravimetric method) based on the Standard Tables of Food Composition in Japan (2020 Edition)

*BS* Bristol stool form scale

Regarding defecation status based on daily records, no significant differences were observed between the two groups for any of the items. Regarding the changes (%) in each constipation symptom based on the Rome IV criteria, Group F showed a significantly greater reduction in straining hard during defecation compared to Group K (*p* = 0.049). Regarding sleep status, Group F showed a significant decrease (improvement) in the PSQI global score compared to Group K (*p* = 0.004), and the score for sleep duration among the seven component scores significantly decreased (*p* = 0.002; Fig. 5). Regarding mental health, Group F showed a significant decrease (improvement) in the PHQ- 9 global score compared to Group K (*p* = 0.009). Additionally the scores for sleep problems (*p* = 0.019) and low energy (*p* = 0.002) among the nine question items significantly decreased (Fig. 6).

Regarding HRQoL, Group K showed a significant increase (improvement) in the physical component summary compared to Group F among the three summary scores (*p* = 0.044). On the other hand, Group F showed a significant increase (improvement) in the role emotional subscale score compared to Group K among the eight subscale scores (*p* = 0.035; Fig. 7). Regarding dietary intake, no significant differences were observed between the two groups for any of the items.

### Comparison within groups before and after the intervention

Comparisons within groups before and after the intervention are shown in Table 6.

**Table 6:**
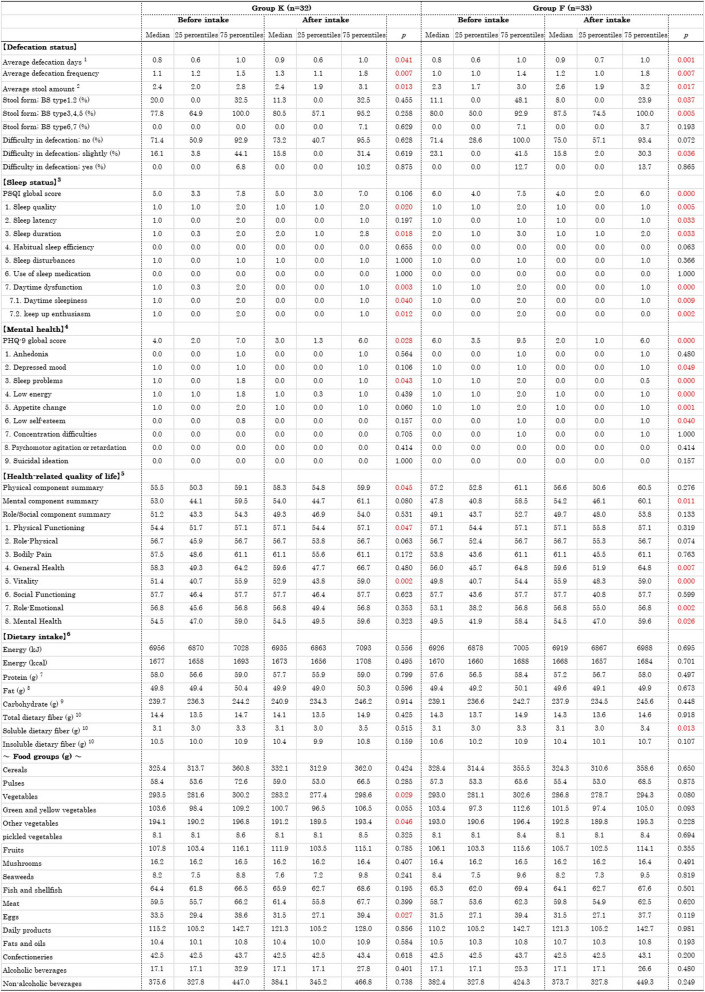
Comparison of within groups before and after the intervention

Using the Wilcoxon signed-rank sum test. Defecation status was analyzed using data from survey A1 and one month’s defecation data aggregated from Surveys A2 and A3. Other comparisons were analyzed using data from the first and third surveys

^1^The data are averages per survey day. All other data for defecation status are averages per defecation day

^2^Number of egg conversion

^3^According to the Pittsburgh Sleep Quality Index (PSQI-J)

^4^According to the Patient Health Questionnaire- 9 (J-PHQ- 9)

^5^According to the Medical Outcomes Study 36-Item Short-Form Health Survey version 2 (SF- 36)

^6^According to the Food Frequency Questionnaires NEXT short version (short-FFQ)

^7^Protiens calculated based on the Japanese amino acid composition table

^8^Fatty acids expressed in triacylglycerol equivalents

^9^The amount of carbohydrates used for energy calculation based on the Standard Tables of Food Composition in Japan (2020 Edition)

^10^The dietary fiber intake was calculated using the modified Prosky method (enzymatic-gravimetric method) based on the Standard Tables of Food Composition in Japan (202 0Edition)

*BS *Bristol stool form scale

#### Defecation status

**Fig. 3:**
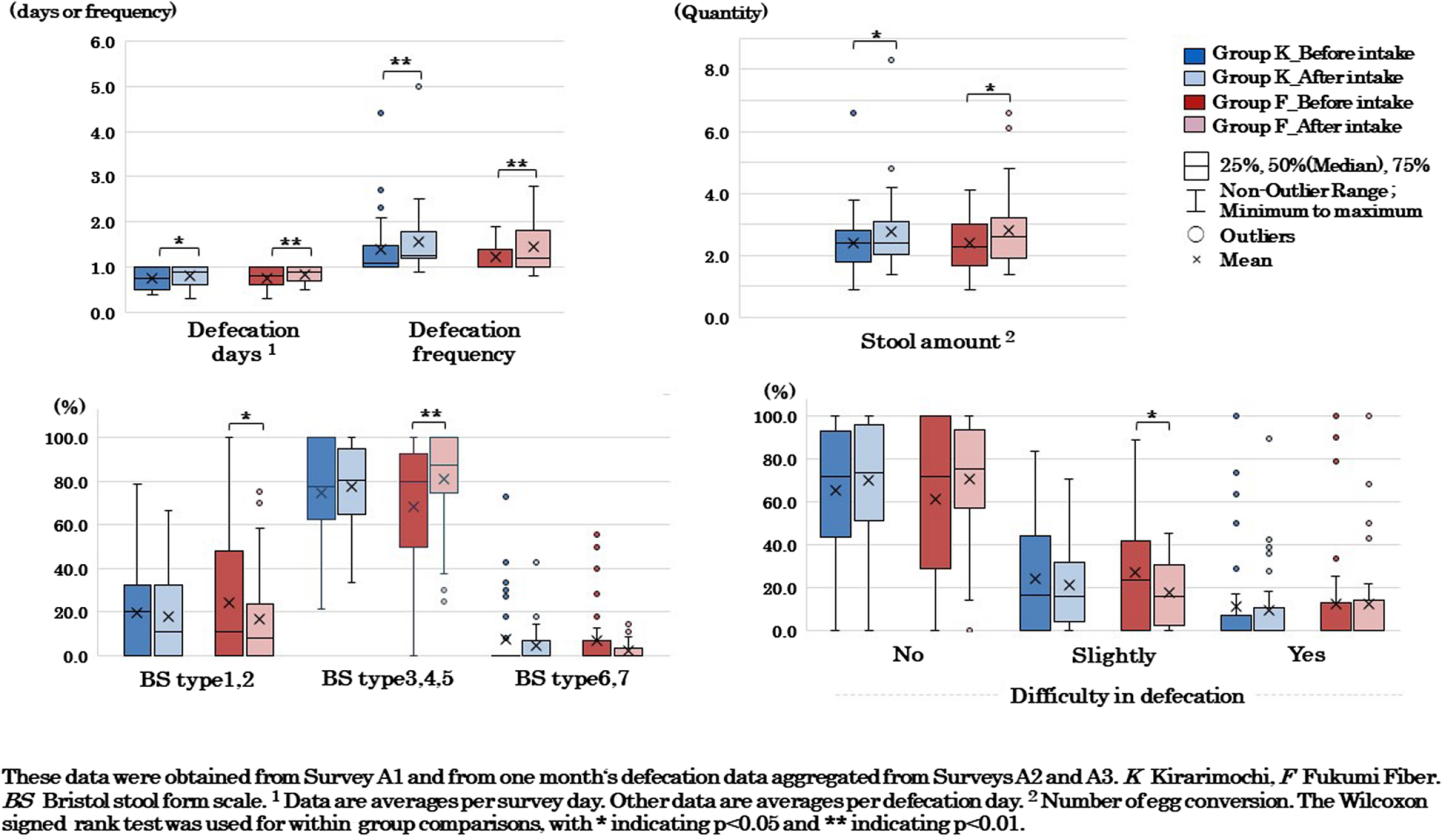
Defecation status before and after the intervention

Comparisons of defecation status showed that in Group K, defecation days (*p* = 0.041), defecation frequency (*p* = 0.007), and stool amount (*p* = 0.013) increased significantly (Fig. 3). In Group F, defecation days (*p* = 0.001), defecation frequency (*p* = 0.007), stool amount (*p* = 0.017), and the percentage of BS types 3, 4, and 5 (*p* = 0.005) increased significantly. Additionally, the percentage of BS types 1 and 2 (*p* = 0.037) and the percentage of slight difficulty in defecation (*p* = 0.036) decreased significantly.

**Fig. 4:**
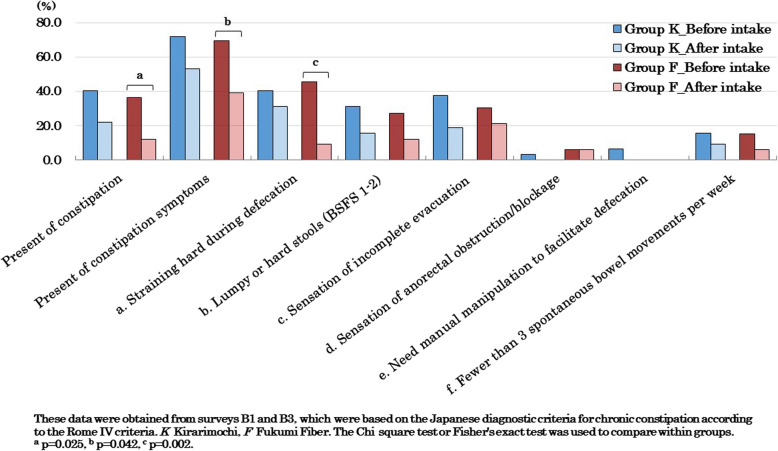
Constipation symptoms before and after the intervention

Regarding the changes in the percentages of each constipation symptom based on the Rome IV criteria, there were no significant changes in Group K for any of the items (Fig. 4). In Group F, the percentage of straining hard during defecation (*p* = 0.002) among the symptoms (a–f) significantly decreased (improved), and there was a significant decrease in both the percentage of constipation symptoms (*p* = 0.025) and the percentage of constipation (*p* = 0.042).

#### Sleep status

**Fig. 5:**
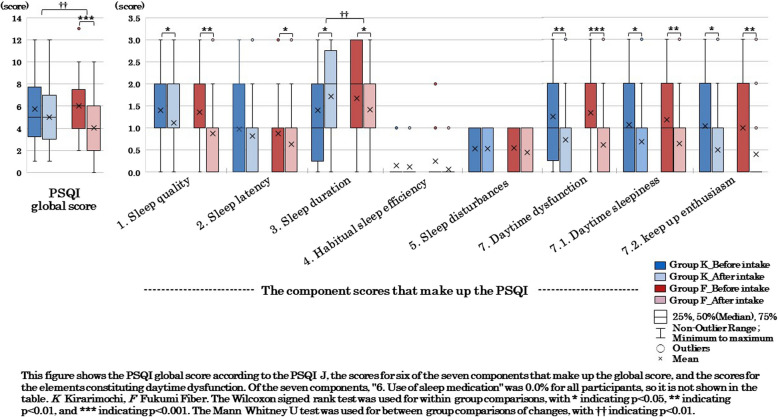
Sleep status before and after the intervention

Comparisons of sleep status using the PSQI-J showed that in Group K, there was no significant change in the PSQI global score, but two of the seven component scores, sleep quality (*p* = 0.020) and daytime dysfunction (*p* = 0.003), significantly decreased (improved) (Fig. 5). Additionally, the scores for daytime sleepiness (*p* = 0.040) and the ability to keep up enthusiasm (*p* = 0.012), which are components of daytime dysfunction, also significantly decreased. On the other hand, the sleep duration score significantly increased (shorter sleep duration) (*p* = 0.018). In Group F, the PSQI global score significantly decreased (improved) (*p* < 0.001), and four of the seven component scores—sleep quality (*p* = 0.005), sleep latency (*p* = 0.033), sleep duration (*p* = 0.033), and daytime dysfunction (*p* < 0.001)—also significantly decreased. Additionally, the components of daytime dysfunction, daytime sleepiness (*p* = 0.009) and the ability to keep up enthusiasm (*p* = 0.002), significantly decreased.

## Mental health

**Fig. 6:**
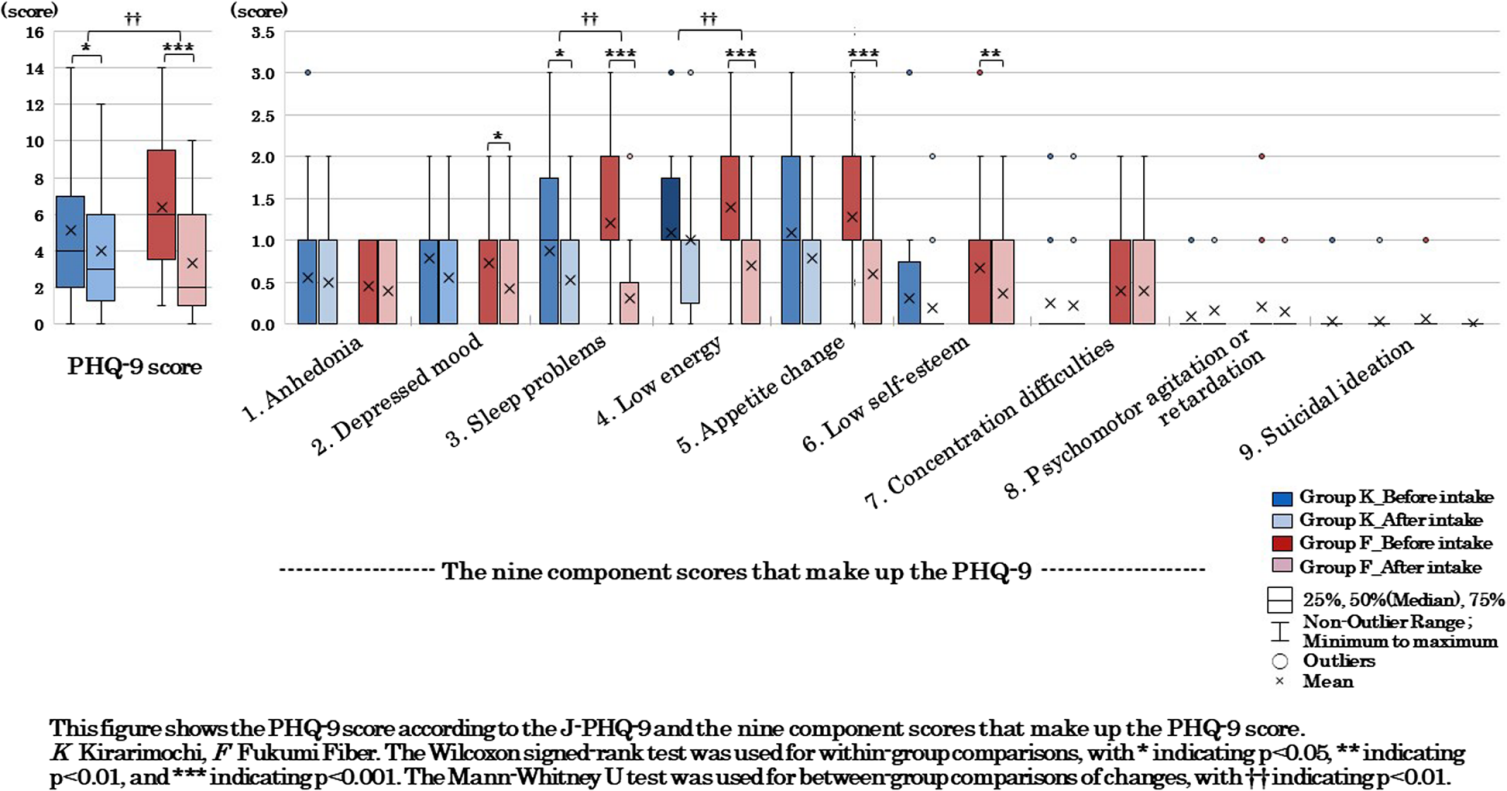
Mental health before and after the intervention

Comparisons of mental health using the J-PHQ- 9 showed that in Group K, the PHQ- 9 score significantly decreased (improved) (*p* = 0.028), and one of the nine questionnaire items, sleep problems (*p* = 0.043), significantly decreased (Fig. 6). In Group F, the PHQ- 9 score significantly decreased (improved) (*p* < 0.001), and five of the nine questionnaire items—depressed mood (*p* = 0.049), sleep problems (*p* < 0.001), low energy (*p* < 0.001), appetite change (*p* = 0.001), and low self-esteem (*p* = 0.040)—significantly decreased.

## HRQoL

**Fig. 7:**
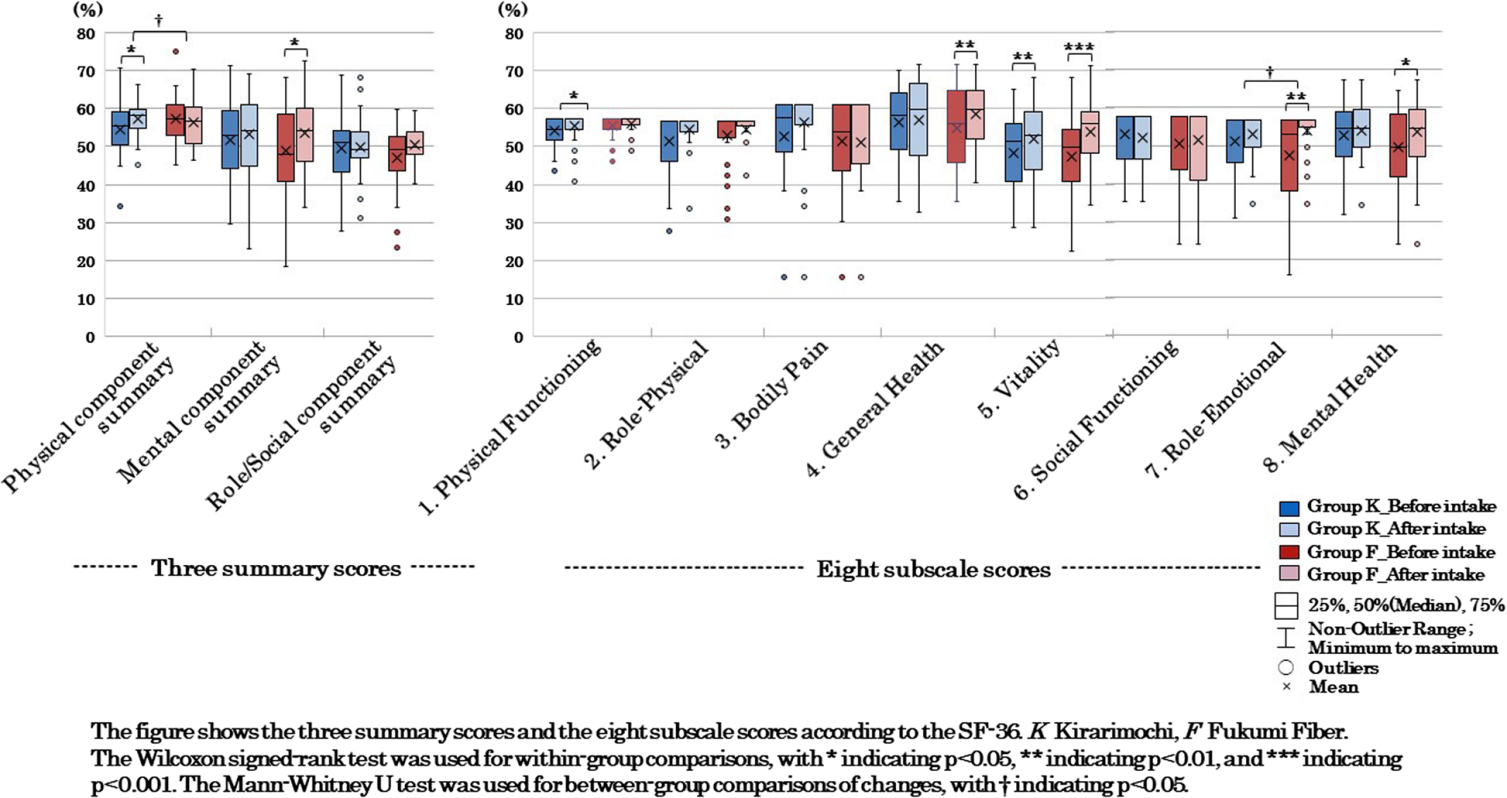
Health-related quality of life (HRQoL) before and after the intervention

Comparisons of HRQoL using the SF- 36 showed that in Group K, the physical component summary significantly increased (improved) (*p* = 0.045) among the three summary scores, and two of the eight subscale scores—physical functioning (*p* = 0.047) and vitality (*p* = 0.002)—significantly increased (Fig. 7). In Group F, the mental component summary significantly increased (improved) (*p* = 0.011) among the three summary scores, and four of the eight subscale scores—general health (*p* = 0.007), vitality (p < 0.001), role emotional (*p* = 0.002), and mental health (*p* = 0.026)—significantly increased.

## Dietary intake

Comparisons of dietary intake using the short-FFQ showed that in Group K, the intake of vegetables (*p* = 0.029), other vegetables (*p* = 0.046), and eggs (*p* = 0.027) among the food groups significantly decreased, but there was no change in DF intake. In Group F, water-soluble DF intake significantly increased. However, there were no changes in the intake of any other food groups (Table 6).

### Multivariate linear regression analysis of changes in constipation symptoms

#### Multivariate linear regression analysis of changes in constipation symptoms

The results of the regression analysis with changes in constipation symptoms as the dependent variable (Table 7) showed that in Model A1, total DF intake was identified as a determinant of changes in constipation symptoms, independent of the type of barley and the frequency of waxy barley rice intake (%), and the days with waxy barley rice intake more than twice a day (%). In Model A2, β-G intake was identified as a determinant. In the model with insoluble DF intake added as an independent variable, no significant determinants were found (no table).

**Table 7:**
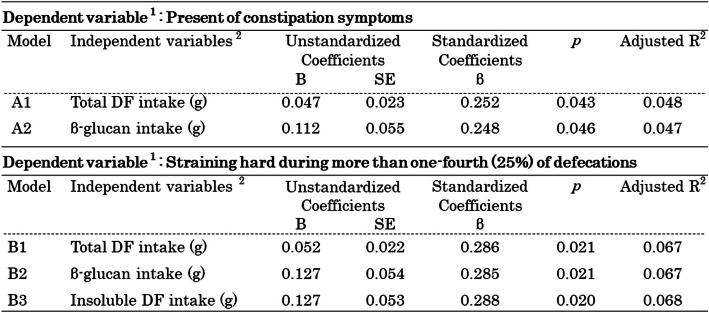
Multiple linear regression analysis

^1^The changes in symptoms before and after the intervention were classified into three categories: “improved,” “unchanged,” and “worsened”

^2^Other independent variables in each model included the type of waxy barley (Kirari Mochi or Fukumi Fiber), the frequency of waxy barley rice intake (%), and the percentage of days with waxy barley rice intake more than twice a day (%)

The results of the regression analysis with changes in “Straining hard during more than one-fourth (25%) of defecations,” an evaluation item for the diagnosis of constipation, as the dependent variable showed that in Model B1, total DF intake was a determinant, independent of the type of barley, the frequency of barley intake (%), and the days with waxy barley rice intake more than twice a day (%). β-G intake was identified as a determinant in Model B2, and insoluble DF intake was identified as a determinant in Model B3.

## Discussion

In this study, healthy young Japanese women consumed waxy barley rice for 4 weeks, which was prepared by mixing rice with two types of waxy barley containing different amounts of β-G. Then, we examined the hypothesis that differences in waxy barley cultivars might result in variations in β-G and DF intake, as well as differing effects on defecation, sleep, mental health, and HRQoL.

As a result, although no differences were observed in the intake status of waxy barley rice between cultivars, significant differences were found in β-G and DF intake. Unexpectedly, while no major differences were observed in the effects of waxy barley cultivars on defecation, the findings suggested that high-β-G waxy barley may have more beneficial effects on sleep and mental health.

### The effects on dietary fiber intake

Significant differences in DF intake were observed among the waxy barley cultivars used in this study. On the other hand, there were no differences in the frequency of waxy barley rice intake (%) or the percentage of days with waxy barley rice intake more than twice a day (%) (Table 4). Therefore, the differences in DF intake are thought to be primarily attributable to the content of DF, including β-G, in waxy barley itself, rather than the consumption patterns of waxy barley rice.

In the trial, participants were encouraged to consume waxy barley rice more than twice a day to achieve a daily β-G intake of 3 g. However, the actual achievement rate was just over 70%, and the average daily consumption frequency was approximately 1.5 times, falling short of the target frequency (Table 4, estimated from the frequency of waxy barley rice intake [%]). Nevertheless, the average Total DF intake from barley was 6.3 g for common waxy barley and 10.7 g for high-β-G waxy barley. These amounts corresponded to approximately 30% and 50%, respectively, of the recommended DF intake for Japanese adults (at least 18 g/day for women and 21 g/day for men) [7]. Therefore, the consumption of waxy barley as waxy barley rice in daily meals is considered to make a substantial contribution to DF intake in both cultivars. On the other hand, if the aim is to achieve β-G intake, it was found that with common waxy barley, the blending proportion and consumption frequency used in this study made it difficult to reach the target amount. When it is difficult to increase the blending proportion or consumption frequency due to individual preferences or daily intake habits, the use of high-β-G waxy barley is considered to greatly enhance β-G intake, facilitating the achievement of the target amount. In this study, the blending proportion was set at 30% to maintain participants’ preferences and ensuring a high intake rate. Among previous studies, there is one that investigated the same cultivar, Kirarimochi, using a blending proportion of 50% [57]. Further studies are required to evaluate how changes in blending proportion affect DF intake.

### The effects on defecation

The effects of DF intake on defecation have been clarified in meta-analyses [19, 22]. Many of these studies utilized processed powders like glucomannan, psyllium, and inulin, and did not include research using barley. However, there are several studies that have investigated the effects of consuming processed barley or whole barley on human defecation. Studies on processed barley products have reported increased fecal weight, reduced fecal pH, and higher butyric acid excretion in Australia [58], alongside improved defecation frequency and stool output in Japanese adults with a tendency toward constipation [59]. As far as we know, there are only two studies on whole barley consumption: one reported an increase in stool volume in young women [60], and the other showed that 30% waxy barley rice (Kirarimochi) improved defecation frequency and decreased days of laxative use in constipated elderly individuals [61]. This study also demonstrated that both waxy barley cultivars led to increases in defecation days, defecation frequency, and stool amount following the intervention (Fig. 3, Table 5), aligning with prior research findings. These findings are considered to suggest the defecation-promoting effects of barley.

In addition, the results of regression analysis indicated that total DF and β-glucan intake were significant factors in improving constipation symptoms, regardless of the waxy barley cultivar. Similarly, for the constipation symptom of straining hard during defecation, insoluble DF, in addition to total DF and β-glucan, was identified as an important factor (Table 7). These findings are considered to support the two mechanisms by which DF prevents and alleviates constipation—namely, the gel-dependent water-holding capacity of soluble DF and the mechanical stimulation of the intestinal mucosa of insoluble DF [24, 62]—and suggest the expected benefits of barley, which contains a balanced composition of both soluble and insoluble DF.

In this study, while several improvements in defecation status were observed within each group for both waxy barley cultivars, differences between the cultivars were noted only in the constipation symptom of straining hard during defecation. Therefore, even common waxy barley, which fell below the target β-G level, may still have a certain effect on promoting defecation.

Previous studies used daily DF and β-G amounts of 1.7 g and 1.0 g [59], as well as 5 g and 3 g [61], respectively, and a Canadian report suggested that increasing DF intake by 1 g per day could reduce constipation-related healthcare costs [63]. Although this study does not allow for specific recommendations regarding effective DF or β-G intake levels, it indicates that a β-G intake of less than 3 g per day (2.5 g of β-G and 6 g of DF in this study) might still have beneficial effects on defecation.

### The effects on sleep, mental health, and HRQoL

As research on the microbiota–gut–brain interaction advances, investigations into the effects of DF on this interaction are also progressing. The 2024 systematic review and meta-analysis [64] found a significant inverse correlation between total DF intake and symptoms of depression and anxiety in observational studies. The intake of dietary fiber has been reported to increase fecal Bifidobacterium and Lactobacillus [65], with the gut microbiota fermenting DF to produce physiologically SCFAs [29], an increase in fecal SCFAs observed with barley consumption [34–36], and associations between certain SCFAs and mood disorders also noted [32].

The significant improvements in mental health observed with high-β-G waxy barley compared to common waxy barley in this study are considered to support these findings.

Additionally, studies using probiotics, including Lactobacillus and Bifidobacterium, have reported improvements in PSQI among healthy adults [66], alleviation of stress symptoms, improvements in depressive mood and sleep quality (PSQI score) in young men [67], as well as improvements in sleep quality (PSQI score) and certain aspects of HRQoL among healthy adults (Patterson et al.) [68]. The significant improvement in the PSQI global score observed with high-β-G waxy barley compared to common waxy barley in this study aligns with these findings and suggests that DF intake may have beneficial effects on sleep. In this study, both groups showed improvements in several items related to sleep evaluation before and after the intervention, as well as improvements in “sleep problems” (a component of the PHQ- 9) in the evaluation of mental health. It is considered that these findings may indicate short-term impacts of sleep on stress, physical health, depression, and QoL [69], as well as the intricate bidirectional relationship between sleep and stress [70].

As far as we have searched, no studies have been found examining the effects of probiotic intake, much less DF, on HRQoL. In this study, the usefulness of high-β-G waxy barley compared to common waxy barley was confirmed for sleep and mental health; however, no such usefulness was observed for HRQoL.

The improvements in vitality (a subscale score of SF36) observed before and after the intervention in both waxy barley cultivars are consistent with the findings of Patterson et al. [68], who also used SF36, and it is notable that the mental component summary improved in high-β-G waxy barley.

### Strengths, limitations, and perspectives

This study is one of the few human trials that have examined the effects of barley on defecation. It is also likely the first report to examine barley's effects on sleep, mental health, and HRQoL. This study clarified not only the total DF intake derived from barley but also the individual DF intake amounts, including β-G and insoluble DF, as well as the overall barley intake. The barley intake in this study was around 40 g for both waxy barley cultivars, an amount likely to be incorporated into realistic dietary practices.

This study has several limitations. We used waxy barley rice, which was prepared by cooking waxy barley grains as the test meal. In essence, it should be noted that in this study, which used whole barley rather than β-glucan extracts, various components in barley [39], including β-glucan, may have contributed to the favorable effects on physiological functions. This suggests the significance of consuming barley as a whole rather than isolating its individual components. Additionally, it should be noted that the participants of this study were Japanese female university students, and the results may vary depending on different age groups, genders, ethnicities, and occupational backgrounds. The study period was conducted during a time with minimal impact from temperature and humidity, and stress periods related to student exams were avoided to minimize the effects on defecation, sleep, and mental health. However, the potential effects of menstruation specific to women and individual stressors that may have arisen were not identified or taken into consideration. Finally, the evaluations of defecation, sleep, mental health, and HRQoL used in this study were mostly based on subjective assessments by the participants. The improvement in subjective indicators is thought to be meaningful; however, it is important to note that these were not objective measures. Due to these limitations, generalizing the results of this study to the broader population is challenging, necessitating further studies in diverse groups to explore the multifaceted health functionalities of barley.

## Conclusions

This study demonstrated that barley, particularly waxy barley, consumed as waxy barley rice, serves as an excellent source of DF. Additionally, this study showed that for the purpose of β-G intake, high-β-G waxy barley is clearly more beneficial. Regarding the health effects of barley, this study suggested that both high-β-G waxy barley and common waxy barley might provide benefits for defecation, even with a blending proportion of 30% and an actual consumption frequency of approximately 1.5 times per day. For sleep and mental health, high-β-G waxy barley may be suggested to have some potential benefits. Reports examining the effects of barley on human health are extremely limited. Further research is needed to explore the diverse potential of barley as a prebiotic.

## Data Availability

No datasets were generated or analysed during the current study.

## Abbreviations

DF: Dietary fiber
QoL: Quality of life
HRQoL: Health-related quality of life
β-G: β-Glucan
EFSA: The European Food Safety Authority
FDA: Food and Drug Administration
SCFAs: Short-chain fatty acids
AOAC: Association of Official Analytical Chemists
PSQI-J: The Japanese version of the Pittsburgh Sleep Quality Index
J-PHQ- 9: The Japanese version of the Patient Health Questionnaire-9
SF- 36: Medical Outcomes Study Short-Form 36-Item Health Survey version2, the Japanese version
short-FFQ: Food Frequency Questionnaires NEXT short version
BS: Bristol stool form scale
